# Primary Cardiac Angiosarcoma

**DOI:** 10.1155/SRCM/2006/39130

**Published:** 2006-10-04

**Authors:** Dhatri Kodali, Kala Seetharaman

**Affiliations:** ^1^Division of Hematology, Oncology and Transplantation, University of Minnesota, 420 Delaware St SE, Minneapolis, MN 55455, USA; ^2^Division of Hematology/Oncology, Worcester Medical Center, Fallon Clinic, Worcester, MA 01608, USA

## Abstract

Primary cardiac sarcoma is a rare clinical entity, with an incidence of 0.0001% in collected autopsy series. The majority of the literature describes a uniformly dismal prognosis with a median survival of only 6 months for these aggressive tumors. Standard surgery, adjuvant chemotherapy, and radiotherapy have been consistently unsuccessful. Early heart transplantation and novel radiation therapy approaches may offer a survival benefit in nonmetastatic tumors, but up to 80% of the patients present with systemic metastasis at diagnosis. Though several chemotherapeutic regimens have been tried, the role of chemotherapy is not well established and outcome data available is minimal. Liposomal doxorubicin (PLD) has been shown to be useful in the treatment of soft tissue sarcomas, and our case supports its use in cardiac angiosarcoma.

## CASE REPORT

**Figure 1:**
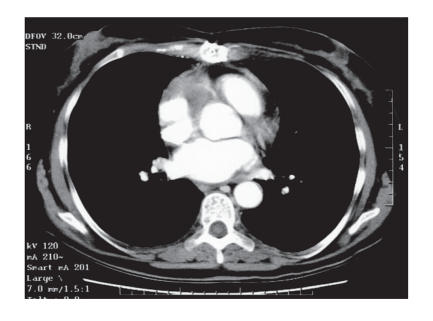
Cardiac mass on CT scan before chemotherapy.

**Figure 2:**
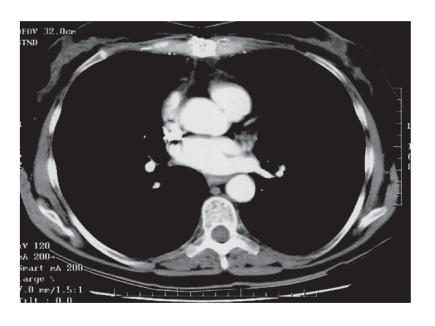
Cardiac mass on CT scan after chemotherapy.

A 66-year old woman presented in July 1999 with 2-week history of flu-like illness and progressive exertional dyspnea. She was found to have a cardiac mass on transesophageal echocardiogram. MRI-magnetic resonance imaging revealed a 3.5/4/4-cm mass in the right atrium and ventricle extending into the anterior superior mediastinum. The patient underwent sternotomy and exploration, and the mass was found to be in the atrioventricular junction. Biopsy revealed a high-grade angiosarcoma. Staging evaluation revealed a pulmonary metastasis in the left base posteriorly. Her performance was 2 on the ECOG scale. After detailed discussion of treatment options, the patient opted for minimal intervention with minimal toxicity. Liposomal doxorubicin has been used in soft tissue sarcoma and is well tolerated; hence she was treated with Doxil 40–50 mg/m2 q 4 weeks for total 11cycles. She had an excellent clinical response after 2 cycles and radiological response after the third cycle (Figures 1, 2). She enjoyed good quality life and had no significant side effects from chemotherapy. However, she relapsed with extensive pulmonary disease after 15 months. Thereafter the patient received two cycles of combination chemotherapy consisting of MAID regimen (mesna, adriamycin, ifosfamide, and dacarbazine) with minimal response. The patient eventually succumbed to metastatic disease 16 months after her initial diagnosis.

## DISCUSSION

Twenty five percent of primary cardiac tumors in adults are malignant. Sarcomas represent the commonest histology, accounting for 20% of all cardiac neoplasms. Still, primary cardiac sarcoma is a rare clinical entity, with an incidence of 0.0001% in collected autopsy series [1]. The low incidence of primary cardiac sarcomas reflects the overall low incidence of sarcomas in the general population and the small percentage of body weight of heart (0.5%) compared with muscle (40%). Angiosarcomas are the commonest cardiac sarcomas and make up 33% of cases.

The majority of cardiac sarcomas occur between the third and fifth decades of life with a male preponderance (M : F ratio 2 : 1). Primary cardiac sarcoma seldom causes symptoms until late in the course. Most common symptoms include dyspnea, chest pain, CHF, palpitation, fever, and myalgia. The clinical presentation is often found to mimic the more common cardiopulmonary diseases, usually valvular heart diseases, although the most frequent presentation is that of right-sided congestive heart failure. Case reports in the literature describe a variety of clinical manifestations which include arrhythmia, vena caval obstruction, pericardial effusion with or without features of tamponade and conduction disturbances. In contrast with benign tumors, usually located in the left atrium, malignant tumors are found almost exclusively in the right heart, particularly in the right atrium.

Most reported series of cardiac sarcomas describe patients with primary cardiac sarcomas and response to treatment and survival is anecdotal. Complete resection of cardiac sarcoma is difficult, in view of the location and extent of involvement. Often tumors are so large at the time of the operation that complete resection cannot be done. Moreover, up to 80% of patients present with distant metastasis at diagnosis [2]. In general, recommendations for the treatment of nonmetastatic cardiac sarcoma include exploration for local control of the primary tumor, to relieve obstructive symptoms and to prolong disease-free survival. Combined modality approach has been reported to be successful in few cases [3].

Cardiac sarcomas generally have a dismal prognosis with a median survival of only 6 months [4]. Applying the general principles of treatment of soft tissue sarcomas occurring anywhere else in the body, the most critical element is complete surgical resection; however, the location itself is more difficult for obtaining an adequate margin of resection. The emphasis is on early detection and diagnosis. Patients with complete resection have a survival of 24 months compared with 10 months in those with incomplete resection [5]. Orthotopic heart transplantation could allow complete resection of cardiac tumors and has been performed in selected patients. However, most are not transplanted because of the high risk of tumor recurrence or metastasis and the possible enhancement of tumor growth by immunosuppressive drugs [6, 7].

The use of radiotherapy is also restricted in many ways. The typical dose of radiation for sarcomas at most sites is 6000 cgy to 6500 cgy following complete resection of the sarcoma. In unresectable lesions the dose of radiation is often increased to 7000 cgy. Such high doses are not well tolerated by the heart. At a dose of 4000 cgy the incidence of pericarditis is approximately 40%. Hyperfractionated radiotherapy (7050 cgy) along with a radiosensitizer (5-iododeoxyuridine) has been shown to eradicate the tumor in a few cases for locoregional control, after surgical resection in nonmetastatic tumors [2].

The role of adjuvant chemotherapy after surgically resected cardiac sarcoma remains controversial. Some of the chemotherapeutic regimens used in the past include DECAV, DTIC, CYVADIC, and VAPAC with variable benefit. There is some evidence to support adjuvant chemotherapy to relieve symptoms and prolong survival as part of the combined modality approach [5]. While other studies, which compared various chemotherapeutic regimes (cyclophosphamide, vincristine, dacarbazine, ifosfamide, methotrexate, vincristine, doxorubicin), concluded that postoperative chemotherapy failed to modify the natural course of patients with resected cardiac sarcomas [8]. There has been an isolated report of a cardiac angiosarcoma that responded to multidisciplinary treatment with recombinant interleukin-2, postoperative chemotherapy, and radiation in spite of incomplete resection [9]. No distant metastasis was noted in the case report.

In our case the patient had a soft tissue mass situated between the right atrium and right ventricle and extending into the anterior superior mediastinum. She also had a pulmonary mass at the left base posteriorly. The patient underwent sternotomy, but the tumor was unresectable. Owing to the metastatic disease on presentation she opted for chemotherapy alone. Since outcome is questionable and therapy is toxic, she preferred to have Doxil therapy alone as opposed to other agents. She had a complete remission after 3 cycles of Doxil therapy. Subsequent CT scans showed more than 90% reduction in the size of her primary tumor and resolution of the pulmonary metastasis. She had a disease- free survival of 11 months. She then recurred with extensive pulmonary metastasis and expired 16 months after initial diagnosis.

The liposomal delivery system has been developed to avoid detection by the reticuloendothelial system (RES) and to increase blood circulation time of doxorubicin. Once inside the tumor, the liposomal covering allows release of the encapsulated doxorubicin. Within the cell, the cytotoxic mechanism of action of doxorubicin is consistent with conventionally delivered doxorubicin. Liposomal doxorubicin may be less susceptible to tumor resistance via the multidrug resistance (MDR) mechanism that is mediated through an overexpression of a P170-glycoprotein than conventional daunorubicin. However, further investigations of PLD in soft tissue sarcoma are necessary [10, 11].

Studies using pegylated liposomal doxorubicin (PLD) in the treatment of sarcomas at other sites have shown variable results in response rates with improved toxicity profile and at least equivalent activity in comparison to doxorubicin [12–14]. However, angiosarcomas in particular have unique histopathological and biologic features compared to other soft tissue sarcomas. PLD has also been used for the treatment of Kaposis sarcoma in AIDS patients [15]. Both Kaposis sarcoma and angiosarcoma are tumors of the endothelial cells and therefore potentially share some common biologic properties [16]. Like angiosarcomas of other organs, atrial angiosarcomas exhibit highly variable histologic patterns, which often overlap those of Kaposi's sarcoma, and may also present metastatic patterns simulating widespread Kaposi's sarcoma. More recently, paclitaxel and liposomal doxorubicin have been reported to have efficacy in angiosarcoma [17, 18]. We hypothesize that our patient might have responded to liposomal doxorubicin because of its unique activity in angiosarcoma in comparison to other sarcomas and potential similarities with Kaposi's sarcoma as described in few other recent studies [19].

## CONCLUSION

Due to the rarity of cardiac angiosarcoma there are no large studies comparing different chemotherapeutic regimens. Several reports have demonstrated that PLD is at least as active as free doxorubicin in soft tissue sarcomas. Notable, a recent article report0 PLD is uniquely active in angiosarcoma. Our current case provides further support for the role of PLD in the treatment of this rare tumor.
